# Editorial

**Published:** 1860-06

**Authors:** 


### EDITORIAL.

From the importance of the subject, and the entire, or at least great inefficiency of the remedies generally used in consumption, much interest has been, and still continues to be felt as to the real value of the hypophosphites in its treatment. Letters and inquiries are constantly addressed to us with respect to the efficacy of the Churchill remedies, as they may very properly be called in the cure of consumption.

The experience of Dr. Richard Quain, in the Brompton Hospital, so entirely accords with our own, that we cannot do better than give a summary of his conclusions as published in the *London Lancet*, for June, 1860. We may be allowed to observe in advance, however, that the great reluctance complained of by inventors, discoverers, and theorists, on the part of the profession to adopt their views, and a determination to sift their assertions in a thorough manner will be fostered by the general results arrived at in the investigations of these highly, we cannot say praised, but vaunted remedies. It is rue in this case the profession had but little to loose in the experiments requisite to arrive at the truth; they would not be required to lay aside reliable remedies, while they were anxiously awaiting the effects of the new, so that there is not much sacrifice made, and I am very sorry that I cannot say there has been no disappointment felt; for from the great assurance with which the claims of the phosphites were put forth, much credence and hope were aroused.

But to the experience of Dr. Quain. He very fairly submitted twenty-two cases of consumption to the hypophosphite course of treatment; two were in the first stage, ten in the second, and ten in the third stage. Of these, six were improved. Of the six, three only to a very slight degree, and only for a short time; in three, improvement was marked, but in one only of the latter has the improvement been permanent; the other two patients both died, notwithstanding the fact that they took them several (over three) months.

Dr. Quain caused abstracts of 22 cases of consumption, treated in the same hospital, by other methods dictated by principles instead of empiricism. Three were in the first stage, five in the second, and fourteen in the third. Sixteen of these were more or less benefitted. He also observed that six of the cases which have been under the hypophosphite treatment without the least benefit, were improved by the other treatment. Dr. Quain remarks in conclusion, that " a review of the preceding facts has led me to form a most unfavorable opinion of the value of hypophosphites in the treatment of phthisis. I believe them to be comparatively, if not absolutely useless. I have been induced to take some little pains in investigating the subject, because of the unhesitating confidence with which their value is asserted and their use recommended in certain quarters; and I have also seen in the cases of some patients who have visited Paris, how much time has been thrown away by substituting the use of these salts for remedies of undoubted efficacy in controlling the progress of phthisis."

---

### TENTH ANNUAL MEETING OF THE ILLINOIS STATE MEDICAL SOCIETY.

The proceedings of the recent Annual Meeting of this Society will be found in this number of the *Examiner.* About fifty physicians were present from different parts of the State, and the two days spent together were devoted diligently to the transaction of the proper business of the Society. Every thing was done pleasantly and in order. We were gratified to find that a much larger number of the Committees were ready to report than for several years past.

If all the reports and papers are published in full, it will make a larger volume of Transactions than any of preceding years. We fear, however, that the Committees of the Society do not yet fully appreciate the nature of the duties they ought to perform. They have very generally adopted the practice of obtaining as many contributions as they can from members of the profession, and then putting them together for a report. Now the object of appointing a Standing Committee on Practical Medicine, Surgery, or Obstetrics, is not to gather up detailed cases of various kinds from different practitioners, and stitch them together in the form of a report, but simply to report, from year to year, such *improvements* as may have been made in the several departments, and the peculiarities that may have been observed in the rise, progress, and causation of epidemic diseases. To do this, they should correspond by circular, or otherwise, with the profession, and gather all the facts they can. But when obtained, they should be carefully sifted, and condensed into one compact report, giving every physician credit, of course, for the facts he furnishes of value.

Such a method would greatly enhance the value, while it would materially lessen the bulk of the reports.

Again, the object of appointing *Special* Committees to investigate particular subjects, is not for the purpose of iliciting essays on the general importance of the topics assigned to them, but to incite to, and develope the results of, special investigations that are likely to add to the actual stock of knowledge on the subject treated of. A report on the general importance of diseases of the eye, or of public hygiene, or of diseases of the chest, may be correct in sentiment and written in excellent style, and yet not contain a single new fact, or new application of old facts. We think when a member of the Society accepts an appointment as special committee on any given subject, he is expected, either by observation, experiment or statistics, or all of these combined, to make some actual addition to the knowledge previously possessed by the profession on that subject. These remarks are not designed to find fault with any of the reports of the recent meeting, any more than those of past years; for so far as we could judge from those read, we think them fully equal in merit to those presented at former meetings. But we would like to see a more active spirit of criticism and original inquiry infused into the reports and doings of our State Society. The physicians and citizens of Paris are entitled to much credit for the judicious arrangements and their unlimited hospitality.

### APOLOGY.

The space occupied by the record of proceedings of the Illinois State Medical Society, and the temporary absence of the Senior Editor, have prevented the insertion of the usual amount of "Clinical Reports," in the present number. These reports will be resumed, however, in our next issue.

---

☞ We learn from a private source that all the Professors of the Ohio Medical College have resigned their places, and a new organization will take place. We hope the Trustees no worse luck than that they may find as efficient and worthy a faculty as the one just resigned.

---

*Another New Medical School.*—We learn from a private letter from our friend Dr. C. A. Logan, of Leavenworth City, that a medical school has been announced there, with the following name: "Medical Department of the Baker University." The faculty is composed of the following gentlemen:—Descriptive and Surgical Anatomy, J. F. Smith, M. D.; Theory and Practice of Medicine,——; Principles and Practice of Surgery, M. S. Thomas, M. D.; Materia Medica and Therapeutics, H. Griffin, M. D.; Chemistry and Toxicology, T. Sinks, M. D.; Institutes of Medicine, W. Booth Smith, M. D.; Medical Jurisprudence and Sanitary Science, G. W. Hogeboom, M. D.; Clinical and Operative Surgery, I. L. Weaver, M. D.; Clinical Medicine, C. J. Lee, M. D. Dr. Logan is the Dean of the Faculty.

---

Upwards of three hundred persons die annually in the *streets* of London, from disease, in addition to deaths from accidents, murders, etc.

---

☞ M. Becqerel, of La Pitie, Paris, has tried the hypophosphites in one hundred cases of phthisis, and declared the remedy useless.

